# RNase H-sensitive accumulation of APOBEC3B in a nucleolus after DNA damage

**DOI:** 10.1042/BSR20253880

**Published:** 2025-10-30

**Authors:** Yohei Saito, Yumi Yamamoto, Fumihiko Yamamoto

**Affiliations:** Division of Radiopharmacy, Faculty of Pharmaceutical Sciences, Tohoku Medical and Pharmaceutical University, Japan

**Keywords:** DNA binding protein, DNA damage response, DNA topoisomerase, nuclear matrix, nucleolus, mutagenesis

## Abstract

Apolipoprotein B mRNA editing catalytic subunit 3B (A3B), a nuclear enzyme that catalyzes cytidine-to-uridine (C-to-U) editing in single-stranded DNA (ssDNA), contributes to genetic diversity in many cancers. A3B is induced or activated by DNA damage owing to a variety of factors; however, the mechanisms by which A3B accesses ssDNA within the genome remain unclear. In this study, we showed that in unstimulated cells, A3B is retained in the nucleoplasm in an RNA-dependent manner. Upon DNA damage induced by camptothecin or actinomycin D (Act D), both targeting topoisomerase I, or by 1-methyl-3-nitro-1-nitrosoguanidine (MNNG), an alkylating agent that generates apurinic/apyrimidinic sites, A3B accumulates at the nucleolar rim and interior. Using confocal microscopy, we assessed the colocalization of A3B with drug-induced R-loops. A3B accumulation was abolished by RNase H treatment, implicating R-loops in its localization. However, the S9.6 antibody, commonly used to detect DNA/RNA hybrids, did not identify R-loop-specific signals in the nucleolus, leaving the direct involvement of R-loops in A3B accumulation unresolved. Conversely, immunoprecipitation-mass spectrometry with data-independent acquisition (IP-MS DIA) revealed increased interactions between A3B and RNA helicases such as DDX17 and DDX21, which are known R-loop-binding proteins, following MNNG or Act D treatment. Our results demonstrate that A3B-induced secondary DNA damage occurs in the nucleolus after DNA damage, providing new insights into the acquisition of cancer diversity involving A3B and the DNA damage response in the nucleolus.

## Introduction

Apolipoprotein B mRNA editing catalytic subunit 3 (APOBEC3) is a cytidine deaminase that not only contributes to defense against viruses but is also a potential mutagen in several cancer types [1,2]. In particular, APOBEC3A (A3A) and APOBEC3B (A3B) play important roles in cancer [3]. APOBEC3 family causes genomic instability by converting cytidine in the genome to uridine, leading to the accumulation of C to T substitutions. Although APOBEC3 family-mediated deamination is absent in normal cells, it is enriched in many primary and metastatic cancers [4,5]. In addition, APOBEC3 family activation correlates with DNA replication stress [6,7]. The APOBEC3 family is generally localized in the cytoplasm or bound to RNA, likely to suppress its cytidine deaminase activity in the genome, as its presence in cells poses a mutational risk [8]. Since A3B is the only member of the APOBEC family that is permanently present in the nucleus, its access to the genome is likely to be severely restricted. However, the underlying mechanisms of its functional regulation and mutagenesis are poorly understood [9].

In Hominoidea, such as humans and apes, A3B is present in the nucleoplasm, and its nuclear localization is speculated to be essential for innate antiviral function [10]. A3B has two DNA-binding sites: the C-terminal DNA-binding site, which has cytidine deaminase activity, and the N-terminal DNA-binding site, which has no enzymatic activity but is similar to the nucleic acid-binding site of A3A and binds to RNA [11]. Nuclear localization of A3B requires an N-terminal domain and residues 15, 19, and 24 for its RNA-binding activity; the RNA-binding activity is not a prerequisite for the nuclear localization of A3B [10]. The APOBEC3 family determines subcellular relocation via RNA-mediated oligomerization, resulting in the formation of ribonucleoprotein particles containing other RNA-binding proteins. For example, A3B in the nuclear matrix binds directly to nuclear matrix components, including hnRNPs, in an RNA-independent manner [12]. These interactions are not fixed and are believed to vary depending on specific circumstances [13].

The nucleus contains a multitude of non-coding RNAs that, together with RNA-binding proteins, form ribonucleoproteins (key components of various nuclear structures), including the nucleolus. For example, RNA-binding proteins involved in pre-mRNA processing, such as the heterogeneous nuclear ribonucleoprotein (hnRNP) family, are predominantly located in the nuclear matrix [14–16]. The nuclear matrix is a filamentous protein network within the nucleoplasm composed of various proteins, such as RNA-binding proteins, structural proteins, and transcription factors, that serves as a site for various physiological functions. Several studies have identified many members of the hnRNP family as candidate A3B-interacting molecules [17]. Consequently, it can be postulated that some A3Bs that form complexes with hnRNPs are present in the nuclear matrix [18]. However, in many previous studies, the detailed nuclear localization of A3B was not addressed because of the general distribution of the protein in the nucleus; it appears to be uniformly distributed in the nucleoplasm and absent or scarce in the nucleolus. Moreover, A3B is excluded from chromosomes during chromosome formation, similar to other common nuclear proteins, which are distributed throughout the cytoplasm during mitosis. Furthermore, factors that alter its nuclear localization and induce DNA binding remain to be elucidated.

Cancer genome analyses suggest that A3B-mediated DNA editing is triggered by its binding to single-stranded DNA (ssDNA) exposed during processes like transcription and replication. Mutations induced by APOBEC3 are often clustered, associated with mismatch repair, and widely distributed across cancer genomes [19]. However, the precise mechanism by which A3B, which is normally repressed, affects the genome remains unclear. Moreover, A3B affects not only spontaneous mutations but also post-irradiation mutations. The change in the number of long deletions after irradiation is particularly pronounced, depending on whether A3B is expressed [20]. This result is attributable to the effect of secondary DNA damage by A3B in addition to the DNA damage response (DDR) to irradiation.

Recently, results from a chromatin immunoprecipitation (ChIP) assay have revealed that A3B binds to the R-loop structure [21]. In addition, the concentration of A3B mutations near DNA break points suggests that the R-loop affected by DNA damage could be a potential target for A3B [22]. Building on this, A3B is speculated to introduce mutations in the ssDNA of the R-loop, similar to activation-induced cytidine deaminase. The overexpression of A3B also affects the formation and resolution of the R-loop [21,23], which is a triple-stranded structure comprising a DNA/RNA hybrid and ssDNA that is formed under various conditions. It plays a role in numerous biological processes, including transcriptional regulation and immunoglobulin class-switch recombination [24]. Additionally, R-loops have been documented at sites of DNA double-strand breaks (DSBs) [25]. Notably, R-loops are affected by ssDNA damage and DSBs. For example, single-strand breaks (SSBs), topoisomerase I (TOPO I)–DNA covalent complexes, and repair intermediates of apurinic/apyrimidinic (AP) sites can form ahead of RNA polymerase (RNAP) during transcription. These structures hinder transcription and promote R-loop formation [26,27]. Actinomycin D (Act D) and camptothecin (CPT) have been demonstrated to stabilize the complexes between TOPO I and DNA in the nucleolus, thereby increasing ssDNA damage and inducing both SSBs and DSBs [28,29]. Similarly, DNA-alkylating agents like 1-methyl-3-nitro-1-nitrosoguanidine (MNNG) activate PARP1, which is abundant in the nucleolus. This not only damages DNA but also RNA, promoting R-loop formation [30–33]. Therefore, although these agents are known to cause DSBs in the nucleoplasm, they may also induce R-loop-associated DNA damage in the nucleolus.

The nucleolus serves as both the site of ribosome biosynthesis and as a stress sensor [34]. The nucleolus is particularly transcriptionally active, and defects in ribosome construction due to DNA damage or RNAP inhibition trigger nucleolar stress responses that regulate cellular stress through structural and functional changes in the nucleolus [35]. The nucleolus consists of a three-layered structure comprising a fibrillar center (FC), a dense fibrillar component (DFC), and a granular component (GC). rRNA is synthesized at the interface between the FC and DFC [36]. Additionally, the nucleolar periphery, designated as the nucleolar rim, has been proposed to represent a fourth subcompartment of the nucleolus [37]. Recently, transcriptional inhibition in the nucleolus or damage to ribosomal DNA (rDNA) has been shown to cause the FCs to fuse and accumulate at the edge of the nucleolus, forming nucleolar caps around the FCs [38]. Mycophenolic acid (MPA), a potent inhibitor of IMPDH, decreases rRNA synthesis [39]. During this process, many nucleolar proteins translocate to the nucleoplasm. A typical example is the migration of the nucleolar protein NPM1 (B23) in response to Pol I inhibition by Act D [40], which has also been observed in cells exposed to various agents such as UV, heat shock, and DNA damage [41–43]. Conversely, Rad9B, which is involved in DNA repair, migrates to the nucleolus in response to UV irradiation and Act D [44]. Abnormal R-loop formation can also contribute to nucleolar stress. For instance, hypoosmotic stress disrupts rDNA and promotes nucleolar cap formation by stabilizing R-loops within the nucleolus [45]. Despite these observations, the molecular mechanisms governing nucleolar stress responses remain unclear. Unlike the well-characterized DDR in the nucleoplasm, DDR in the nucleolus is still poorly understood [46].

The purpose of this study was to elucidate the mutagenic mechanism of A3B in cancer cells, focusing on examining whether A3B can cause DNA-binding that results in secondary DNA damage.

## Results

### A3B bound to nuclear matrix and was not present in the nucleolus

**Figure 1:**
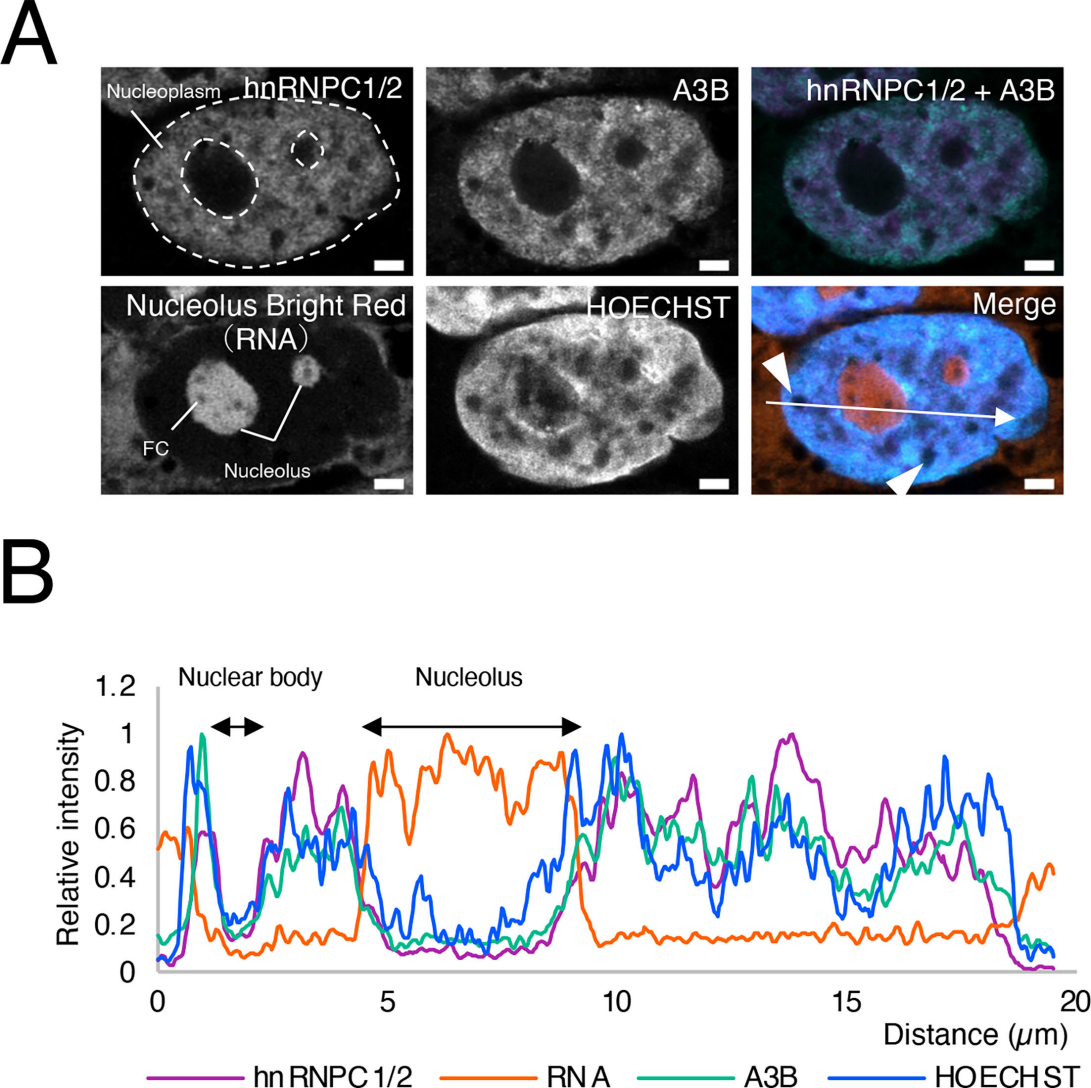
A3B bound to the nuclear matrix and was not present in the nucleolus. (**A**) A3B-AcGFP-expressing cells (green) were fixed with 4% PFA and stained with anti-hnRNPC1/2 antibody (purple), Nucleolus Bright Red (red), and HOECHST (blue). The top right image (hnRNPC1/2 + A3B) shows the merged image of hnRNPC1/2 and A3B staining. The bottom right image shows all merged images. The area outlined in dotted-lines indicates the nucleoplasm. FC, fibrillar center. Arrowheads show nuclear bodies. (**B**) Fluorescence intensity profiles of hnRNPC1/2 (purple), A3B (green), RNA (red), and HOECHST (blue) represent the distribution of signals along the line indicated in the merged image in (**A**). Scale bar = 2 µm.

Super resolution images of the nuclei in HepG2 cells expressing A3B-AcGFP were obtained via confocal microscopy to observe the subcellular localization of A3B (Figure 1). In our study, hnRNPC1/2, as the nuclear matrix marker, was unevenly distributed in the nucleoplasm, avoiding the nucleolus (Figure 1A, top right). Similarly, A3B-AcGFP was also distributed in the nucleoplasm, avoiding the nucleolus (Figure 1A, top center). Moreover, some areas of the nucleus where hnRNPC1/2 and A3B-AcGFP were not present were not stained by either HOECHST or Nucleolus Bright Red, which stains rRNA in the nucleolus, suggesting the presence of a variety of nuclear bodies (Figure 1A bottom right, arrowheads). Furthermore, hnRNPC1/2 and A3B-AcGFP were not lost from the nucleus following membrane permeabilization or DNase I treatment (S-Figure 1). However, these proteins were lost after RNase A treatment. This suggests that A3B is retained in the nuclear matrix via RNA or other RNA-binding proteins, such as the hnRNP family. The mean intensities of hnRNPC1/2 in the nuclei were 1,540.70 ± 772.34 for the control group, 127.63 ± 93.67 for the RNase A treatment group, and 1,025.40 ± 711.41 for the DNase I treatment group. The mean intensities of A3B in nuclei were 231.31 ± 105.80 for the control group, 44.69 ± 9.43 for the RNase A treatment group, and 560.76 ± 269.09 for the DNase I treatment group.

### Accumulation of A3B in the nucleolus due to DNA damage

**Figure 2:**
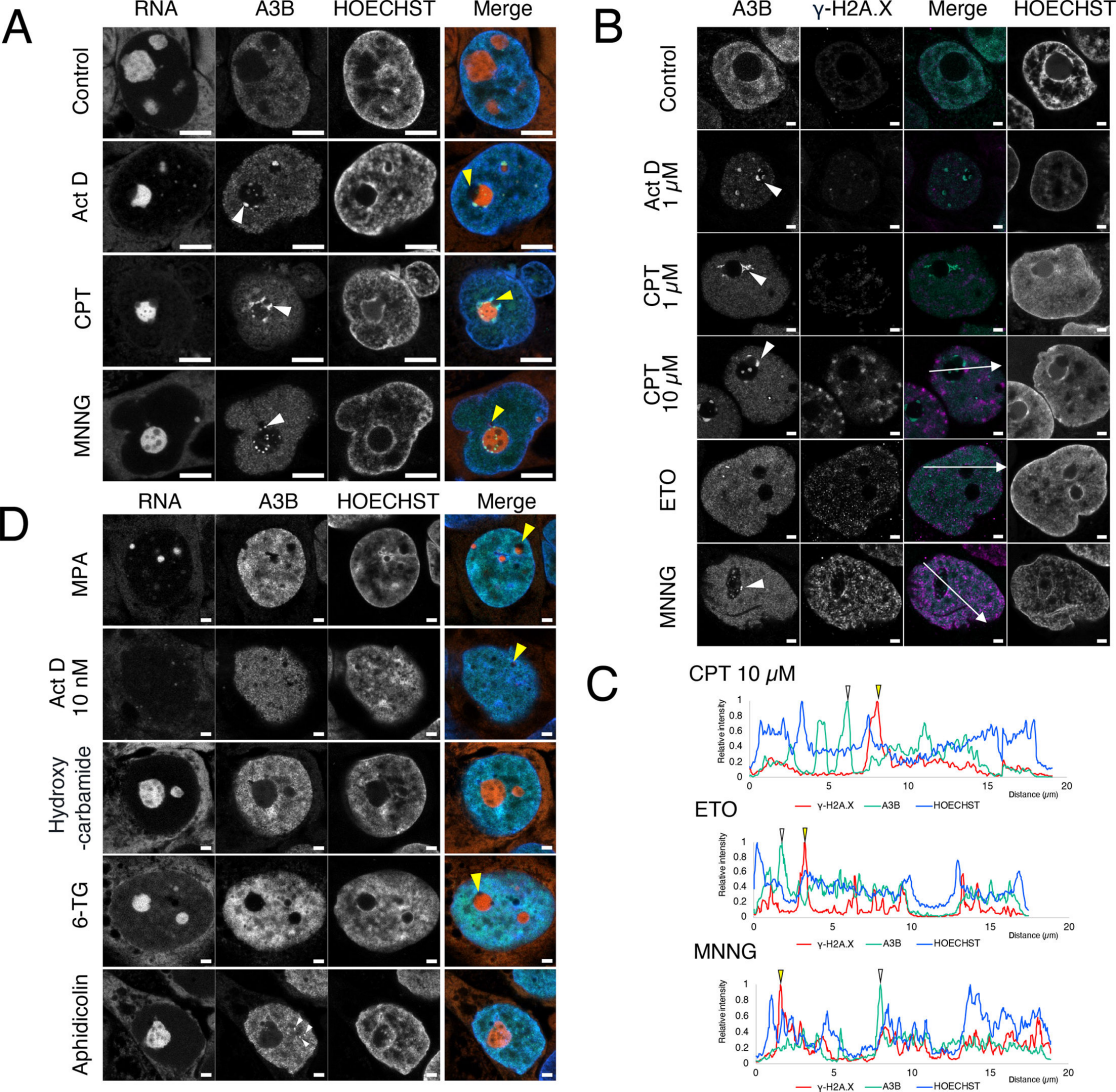
Partial A3B accumulation in the nucleolus after act D, CPT, and MNNG treatment. (**A, D**) A3B accumulated in the nucleolus after DNA damage. A3B-AcGFP-expressing cells (green) were incubated with Act D (1 µM), CPT (1 µM), or MNNG (50 µM) as shown in Figure A, or MPA (10 µM), Act D (10 nM), hydroxycarbamide (0.5 mM), 6-TG (20 µg/ml), or aphidicolin (30 µM) as shown in Figure D for 24 h. The cells were fixed with PFA and stained with nucleolus bright red (red) and HOECHST (blue). The merged images are shown as indicated. Yellow arrowheads indicate FCs, and white arrowheads indicate A3B accumulation. Scale bars: 5  µm (**A**) and 2  µm (**D**). (**B**) A3B-AcGFP cells were treated with Act D (1 µM), CPT (1 µM or 10 µM), ETO (50 µM), or MNNG (50 µM) for 3 h and stained with anti-γ-H2A.X antibody- Alexa Fluor 594 (magenta) and HOECHST (blue). Arrowheads indicate A3B accumulation. Scale bar = 2 µm. (**C**) Fluorescence intensity profiles for γ-H2AX (magenta), A3B (green), and HOECHST (blue) show the distribution of fluorescence along the line indicated in the merged image in (**B**). Yellow arrowheads indicate the peak of γ-H2AX, and white arrowheads indicate the peak of A3B accumulation.

In the present study, we induced DNA damage using various drugs and observed changes in the distribution of A3B to determine whether A3B in the nuclear matrix binds to the site of γ-H2AX or accumulates at a different location as DNA damage occurs. The DNA-alkylating agent MNNG, the DNA intercalator Act D, and the TOPO I inhibitor CPT were identified as agents responsible for altering the localization of A3B. Addition of the three drugs initiated A3B accumulation in the nucleolus within 15 min (S-Figure 2A). However, after 3 h, A3B levels increased in the periphery of the nucleolus in Act D- and CPT-treated cells, and in the interior of the nucleolus for MNNG-treated cells (Figure 2A). MNNG generated γ-H2AX foci throughout the nucleus except in the nucleolus (Figure 2B and C). The number of γ-H2AX foci increased only slightly under the influence of Act D (1 µM) and CPT (1 µM) for a period of 3 h. In contrast, the TOPO II inhibitor etoposide (ETO) induced γ-H2AX foci; however, no accumulation of A3B was observed in the nucleolus. Furthermore, no accumulation of A3B was observed in the nucleoplasm at γ-H2AX foci created by the addition of ETO, CPT (10 µM), or MNNG. These findings suggest that DDR marked by γ-H2AX is not sufficient to recruit A3B.

### Nucleolar stress did not induce A3B accumulation

The addition of MNNG, Act D, or CPT induced nucleolar stress responses, including the fusion of FCs and the appearance of RNA-free nucleolar caps within the nucleolus (Figure 2A, yellow arrowhead). Additionally, A3B accumulated at the rims of the nucleolar caps and nucleoli following drug addition (Figure 2A, white arrowhead; S-Figure 2B and C). Subsequently, we induced nucleolar stress by inhibiting rRNA synthesis and examined its impact on A3B accumulation to ascertain whether nucleolar stress-induced structural alterations in the nucleolus, such as FC migration to the periphery of the nucleolus, contribute to A3B accumulation (Figure 2D). MPA and low concentrations of Act D (10 nM) inhibited rRNA synthesis and induced nucleolar stress. Both MPA and Act D reduced the size of the nucleoli, particularly the RNA-rich DFC and GC (Figure 2D, yellow arrowheads), but did not induce A3B accumulation. The antimetabolite 6-thioguanine (6-TG) inhibited DNA/RNA synthesis, resulting in the appearance of nucleolar caps (Figure 2D, yellow arrowheads); however, no accumulation of A3B was observed. Although the addition of the DNA polymerase inhibitor aphidicolin resulted in a slight accumulation of A3B in the nucleoplasm (Figure 2D, white arrowheads), the DNA synthesis inhibitor hydroxycarbamide did not induce A3B accumulation. These findings suggest that even though the introduction of Act D, CPT, or MNNG promotes nucleolar stress responses, including the development of nucleolar caps and alterations in nucleolar structure, these responses do not result in the accumulation of A3B.

### RNase H-sensitive A3B accumulation

**Figure 3:**
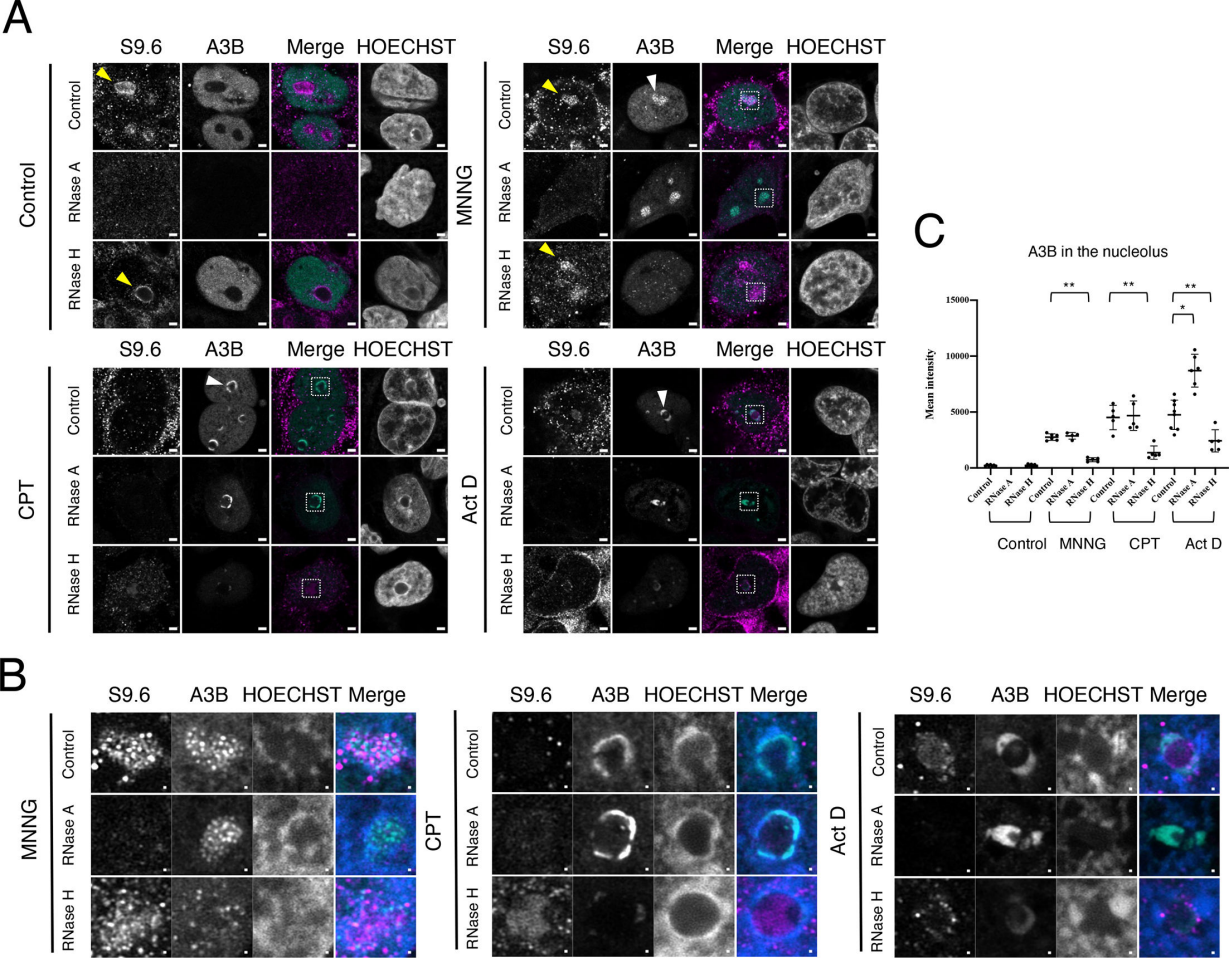
MNNG, CPT, and Act D induced RNase H-sensitive A3B accumulation, but the S9.6 signal did not recognize the R-loop in the nucleolus. (**A**) A3B-AcGFP-expressing cells (green) were incubated with CPT (1 µM), MNNG (50 µM), or Act D (1 µM) for 3 h, treated with RNase A or RNase H, fixed with methanol, and stained with anti-DNA/RNA hybrid antibody (S9.6, rabbit IgG). The cells were subsequently stained with anti-rabbit IgG conjugated with Ax594 (magenta) and HOECHST (blue). The merged images show the merging of S9.6 and A3B. Yellow arrowheads indicate S9.6 signal accumulation, and white arrowheads indicate A3B accumulation. Scale bar = 2 µm. (**B**) Enlarged image of dotted-line box of A. White arrowheads indicate A3B accumulation. Scale bar = 0.2 µm. (**C**) A3B staining intensity in the nucleolus of each drug-treated cells was quantified. Error bars represent mean ± S. D. (*n* = 5–7). *P<0.05 and **P<0.01.

We investigated whether drug-induced accumulation of A3B in the nucleolus is also eliminated by RNase A treatment before cell fixation. In untreated control cells, RNase A eliminated A3B from the nucleoplasm, consistent with previous observations (S-Figure 1C; Figure 3A). However, RNase A treatment did not reduce A3B accumulation in the nucleolus of drug-treated cells (Figure 3B and C). By contrast, treatment with RNase H before fixation effectively removed A3B from the nucleolus in cells treated with MNNG, CPT, or Act D. This indicates that the accumulation of A3B in the nucleolus is dependent on the formation of DNA/RNA hybrid structures such as R-loops, although the pattern of A3B accumulation differed between the drugs. Additionally, RNase H treatment following methanol fixation did not completely eliminate A3B accumulation (S-Figure 3A and B). This outcome could be attributed to the denaturation and aggregation of the protein complex containing A3B resulting from fixation.

**Figure 4:**
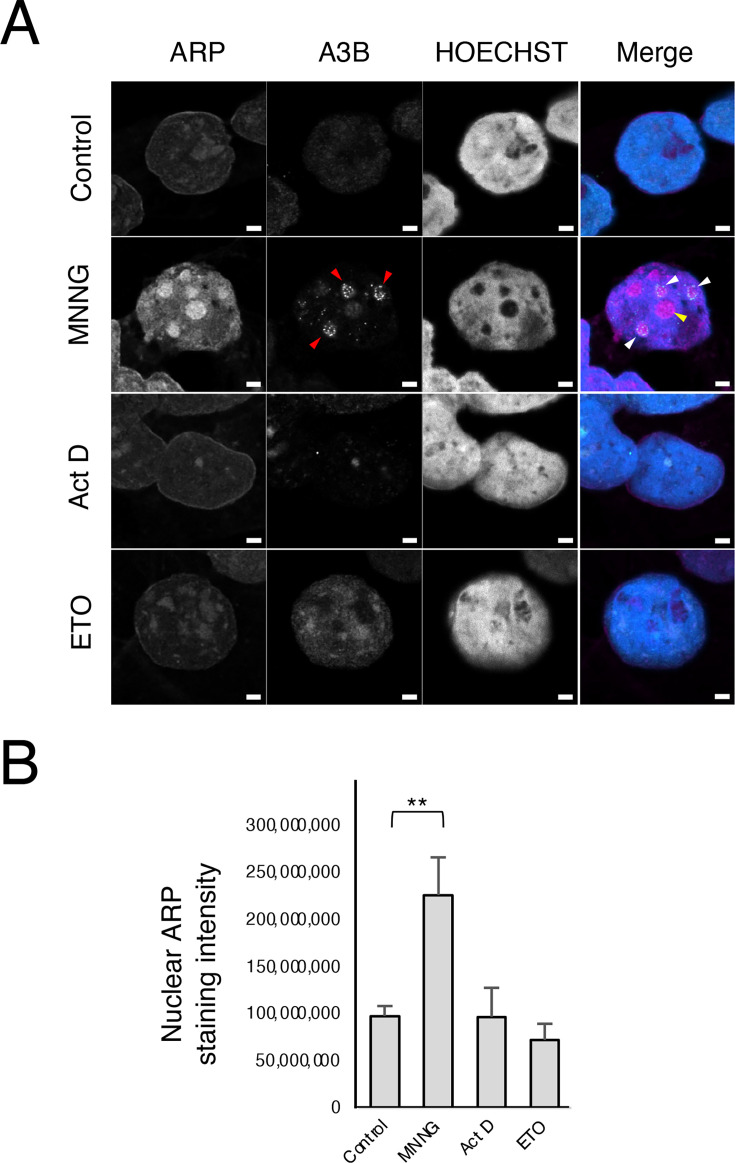
MNNG generates AP sites but Act D and ETO do not. (**A**) A3B-AcGFP-expressing cells (green) were incubated with MNNG (50 µM), Act D (1 µM), or ETO (50 µM) for 3 h. The cells were treated with RNase A, fixed with methanol, blocked with avidin/biotin blocking, incubated with ARP for 1 h, and stained with streptavidin-Ax647 (magenta) and HOECHST (blue). The merged images are shown as indicated. Red arrowheads indicate A3B accumulation. White arrowheads indicate A3B-ARP co-accumulation. Yellow arrowheads indicate ARP accumulation. Scale bar = 2 µm. (**B**) ARP staining intensity in the nucleus of each drug-treated cells were quantified. Error bars represent mean ± S.D. (*n* = 3–4). **P<0.01.

Next, we examined whether the S9.6 antibody, widely used for detecting R-loops in the nucleoplasm by recognizing DNA/RNA hybrids, recognizes R-loops in nucleoli. A robust S9.6 signal was discerned in the nucleoli of control and MNNG-treated cells (Figure 3A, yellow arrowheads), which was not negated by RNase H treatment but was effectively abolished by RNase A treatment. The observation that RNase H-sensitive A3B accumulation persists following RNase A treatment indicates that the DNA/RNA hybrids do not undergo degradation under these conditions. Notably, the absence of a prominent S9.6 signal in the nucleolus after RNase A treatment suggests that the S9.6 signal does not account for the R-loop immunofluorescence typically detected by S9.6 staining. Furthermore, the S9.6 signal in the nucleolus was diminished after 3 h of CPT and Act D treatment (Figure 3A). The reduction of the S9.6 signal in the nucleolus was observed as early as 5 min after the addition of CPT (S-Figure 4). Conversely, the accumulation of A3B was not observed at the 5 min mark following CPT treatment but was observed at the 20 min mark, at which time the S9.6 signal in the nucleolus had almost disappeared. However, because the nucleolus was stained with Nucleolar Bright Red and rRNA was not lost even 3 h after the addition of the drug, no direct relationship was established between the decrease in S9.6 signal intensity and the amount of rRNA (Figure 2A).

**Figure 5A:**
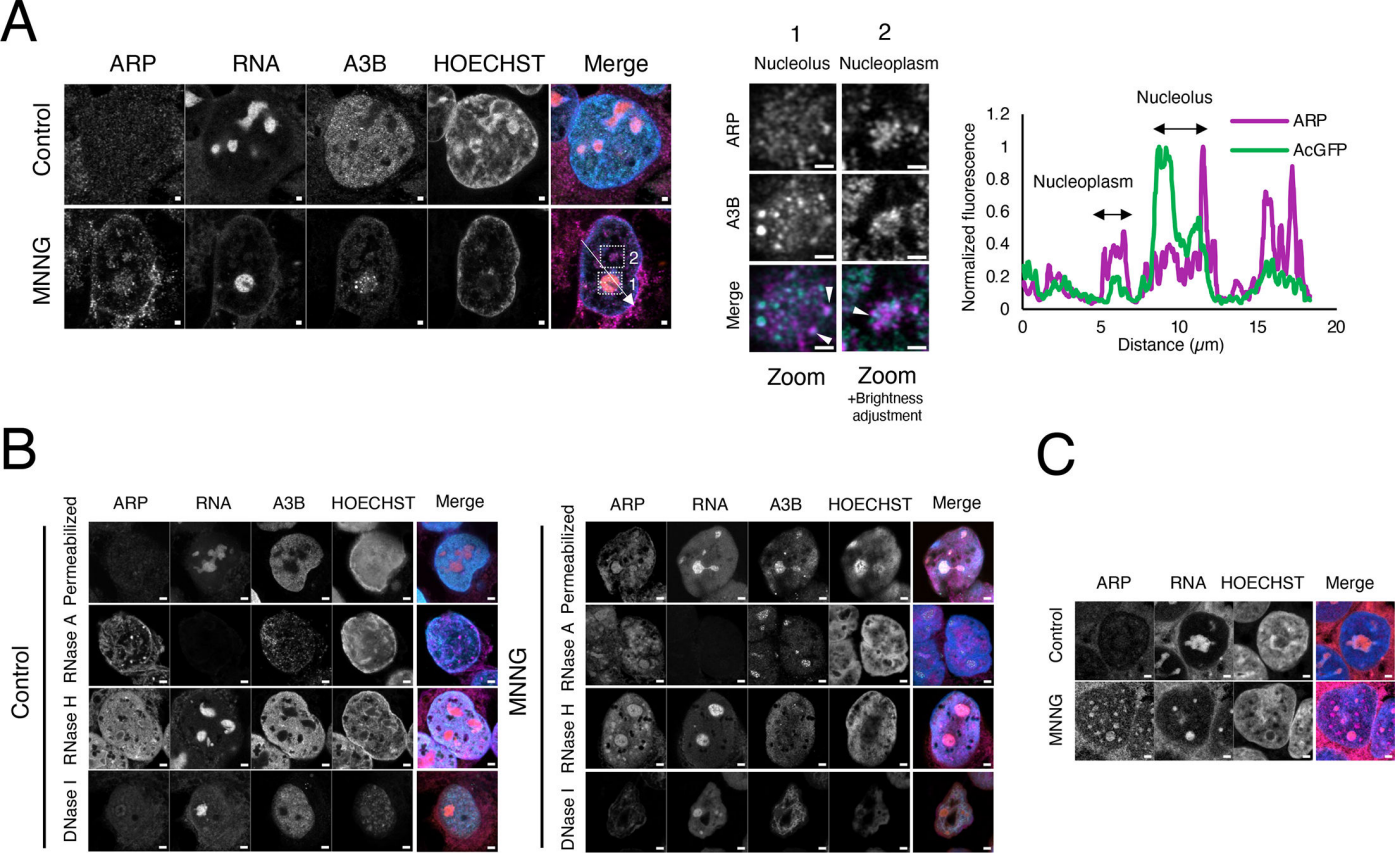
A partial overlap between MNNG-induced AP site and A3B. (**A**) A3B-AcGFP-expressing cells (green) were incubated with or without MNNG (50 µM) for 3 h. The cells were fixed with methanol, blocked with avidin/biotin, incubated with ARP for 1 h, and stained with Streptavidin-Ax647 (magenta), Nucleolus Bright Red (red), and HOECHST (blue). The merged images are shown as indicated. Arrowheads indicate A3B accumulation. The right images show an enlarged image of the dotted-line box of the left images. A fluorescence intensity profile in A3B (green) and ARP (magenta) shows the distribution of fluorescence across the line in the merged image. Scale bar = 1 µm. (**B**) A3B-AcGFP-expressing cells (green) were incubated with MNNG (50 µM) for 3 h. The cells were treated with RNase A, RNase H, or DNase I, then fixed with methanol. The cells then were blocked and stained as in (**A**). Arrowheads indicate A3B accumulation. Scale bar = 2 µm. (**C**) A3B KO cells were incubated with MNNG (50 µM) for 3 h, fixed with methanol, blocked with avidin/biotin, incubated with ARP for 1 h, and stained with Streptavidin-Ax647 (magenta), Nucleolus Bright Red (red), and HOECHST (blue). The merged images are shown as indicated. Scale bar = 2 µm.

Instead of methanol fixation, paraformaldehyde (PFA) fixation was used, allowing detection of the S9.6 signal. After PFA fixation, the S9.6 signal in the nucleolus was relatively weak, likely due to the significantly reduced intensity of the S9.6 signal in the nucleus (S-Figure 5). Conversely, the addition of MNNG resulted in the appearance of the S9.6 signal within the nucleolus, which was not eliminated by RNase H, indicating that it did not originate from the DNA/RNA hybrid (S-Figure 5, red arrowheads). Additionally, S9.6 signal accumulation was observed in cells treated with MNNG and CPT following RNase A treatment (S-Figure 5, yellow arrowheads). However, this accumulation was not present in the control cells and was therefore deemed an artifact. Thus, reliable detection of R-loops in the nucleolus using S9.6 remains difficult.

Although the accumulation of A3B was RNase H-sensitive, which strongly suggests the involvement of the R-loop, the colocalization of A3B and the R-loop could not be confirmed by immunofluorescent cell staining using the S9.6 antibody.

### MNNG generated AP sites in the nucleolus

**Figure 6:**
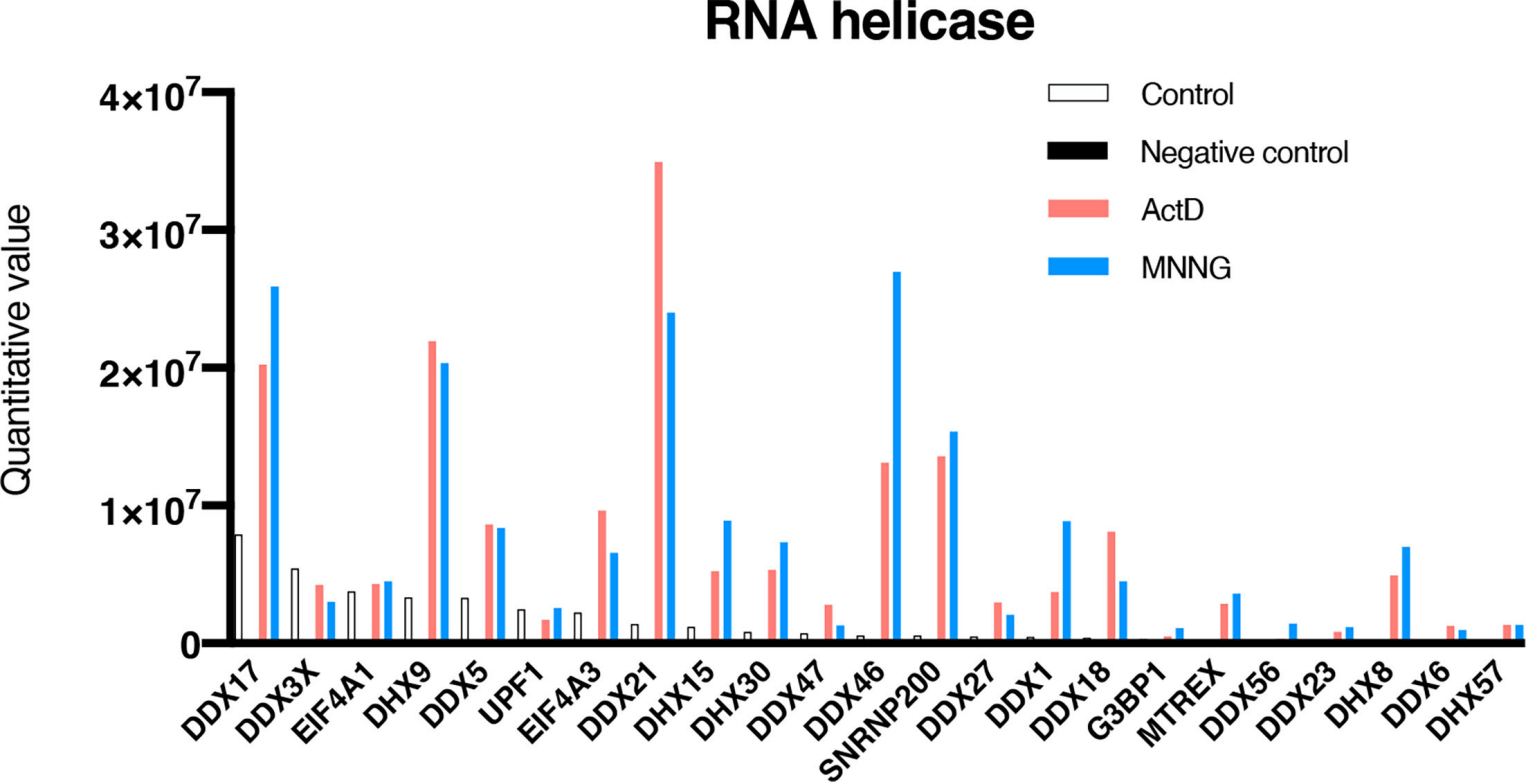
Changes in the quantitative values of RNA helicase. IP-MS DIA analysis of proteins interacting with A3B after DNA damage by act D (1 µM) or MNNG (50 µM) using A3B-AcGFP-expressing cells and AcGFP-expressing cells as control.

We conducted experiments using an aldehyde reactive probe (ARP, N-(aminooxyacetyl)-N’-biotinylhydrazine) to ascertain the presence of MNNG-induced AP sites in the nucleolus. First, DNA was extracted from the MNNG-treated cells, solidified on a plate, treated with ARP, and stained with streptavidin-Alexa 647 (SA-Ax647). The increase in the number of AP sites in the DNA induced by MNNG was discernible via confocal microscopy (S-Figure 6). Subsequently, we investigated the potential use of ARP in cell staining. The effect of RNA on ARP staining to DNA AP sites was compared between RNase A-treated and untreated cells (S-Figure 7A and B). The untreated control cells exhibited low-intensity ARP staining throughout. Conversely, MNNG addition not only increased the intensity of ARP staining throughout the cells but also caused areas of high staining intensity to appear in the nuclei. Conversely, the application of RNase A resulted in a reduction in the overall intensity of ARP staining compared with that in RNase A-untreated control cells. Moreover, the MNNG-treated cells exhibited a distinct nuclear staining pattern in the presence of high-intensity areas, whereas the SA-Ax647 cells showed minimal staining. A histogram of ARP staining intensity per pixel (35 nm × 35 nm) in the nucleus from the confocal microscopy image in S-Figure 7A demonstrates that, irrespective of RNase A treatment, ARP staining intensity in the nucleus was higher in MNNG-treated cells than that in control cells (S-Figure 7B). Additionally, the MNNG-treated cells also exhibited two peaks (the higher peak signified the accumulation of ARPs in the nucleus), indicating the usefulness of ARP staining in vitro, regardless of RNase A treatment, although an increase in background and extranuclear staining should be considered in the case of RNase A-untreated cells.

The ARP staining intensity per nucleus was quantified in cells treated with MNNG, Act D, or ETO after membrane permeabilization and RNase A treatment (Figure 4A and B). Act D served as a negative control, inducing A3B accumulation via TOPO I inhibition but not generating AP sites. Similarly, ETO, an inhibitor of TOPO II, induced DSBs but neither triggered A3B accumulation nor generated AP sites. Some ARP accumulations exhibited colocalization with A3B accumulation in MNNG-treated cells, whereas others demonstrated ARP accumulation with minimal amounts of A3B. Act D-treated cells showed A3B accumulation in areas with high ARP staining intensity. However, the overall nuclear ARP staining intensity in Act D-treated cells did not differ from that in control cells (Figure 4B). ETO-treated cells did not exhibit increased ARP staining intensity or A3B accumulation.

Subsequently, cells were fixed without RNase A treatment to determine whether the MNNG-induced accumulation of AP sites occurred in the nucleolus (Figure 5A). In contrast with the results shown in Figure 4A, the ARP staining intensity was markedly elevated outside the nucleus owing to the application of RNase A treatment prior to fixation. However, following MNNG treatment, ARP staining showed notable accumulation within the nucleolus and nucleoplasm within the nucleus. Most A3B accumulated in the nucleolus (Figure 5A, dotted line, Box 1), while slight A3B accumulation occurred in the nucleoplasm (Figure 5A, dotted line, Box 2). Given the low accumulation of A3B in the nucleoplasm, it was postulated that the site of ARP accumulation with low A3B accumulation observed in Figure 4A was likely the nucleoplasm. MNNG-induced ARP staining in the nucleolus was not eliminated by RNase A or RNase H treatment but was effectively suppressed by DNase I (Figure 5B), suggesting that the ARPs mainly recognized AP sites on DNA. Moreover, DNase I treatment eliminated A3B accumulation, likely because DNase I is capable of cleaving ssDNA, dsDNA, and DNA/RNA hybrids. These findings suggest that MNNG induced the formation of AP sites in the rDNA of the nucleolus, and A3B accumulated in the vicinity of these sites.

Subsequently, we examined whether ARP staining by MNNG increased in A3B KO cells to ascertain whether the AP sites were generated by the action of MNNG or by accumulated A3B. The results demonstrated that MNNG enhanced ARP staining in the nucleolus and nucleoplasm of A3B KO cells (Figure 5C). Moreover, this did not eliminate the possibility of AP site generation by A3B; rather, it indicates that A3B accumulated in the regions where AP sites were generated by MNNG.

### A3B formed a complex with RNA helicase after DNA damage

We performed immunoprecipitation-mass spectrometry data-independent acquisition (IP-MS DIA) analysis using A3B-AcGFP-expressing cells or AcGFP-expressing cells as controls to determine whether changes in sublocalization of A3B after DNA damage by MNNG or Act D can be detected as changes in A3B-interacting proteins. A threshold of five times the protein quantification value observed in AcGFP-expressing cells was established. In total, 92 proteins exhibited a protein quantification value that increased more than ten-fold above the threshold in the MNNG-treated cells, whereas 84 proteins were detected in the Act D-treated cells. This was tabulated based on the molecular function in Gene Ontology (GO) using STRING ver. 12 (Table 1). Strength represents the magnitude of the enrichment effect on unstimulated A3B-AcGFP-expressing cells, and the false discovery rate (FDR) is the p-value of the multigroup analysis using the Benjamini–Hochberg procedure. The top GO terms common to both drugs were ‘U5 snRNA binding’, ‘snRNA’, and ‘RNA helicase’. MNNG-specific terms included ‘adenosylhomocysteinase activity’, which is associated with DNA methylation regulation, while ‘snoRNA binding’ was uniquely enriched following Act D treatment. Of the proteins detected, 60/92 for MNNG and 58/84 for Act D were RNA binding proteins (intensity = 0.89 or 0.91), indicating that RNA helicase active proteins (intensity = 1.40 or 1.39) are also enriched. Therefore, the change in the quantification value of RNA helicase detected before and after the addition of the drug, regardless of the size of the protein quantification value, was examined in the results of the LC-MS/MS DIA analysis. As shown in Figure 6, the helicase on the left side was more highly expressed in the unstimulated control. The localization of these RNA helicases is shown in Table 2. Following the addition of MNNG and Act D, the levels of various RNA helicases, including DDX1, DDX17, and DHX9, which contribute to the resolution of R-loop formation, increased in the A3B coprecipitated proteins. DDX21 and DDX46, which are abundant in the nucleus, were among the RNA helicases with increased quantification, particularly after drug addition (Figure 6). In contrast, cytoplasmic RNA helicases DDX3X, EIF4A1, and UPF1 showed low levels of quantification and minimal alterations. These results indicate that after DNA damage, A3B translocates into a subcompartment rich in proteins with important functions in RNA metabolism, including RNA helicases, in the nucleus. Our observations indicate that DNA damage triggers the introduction of A3B mutations, suggesting high levels of DNA damage in cancer cells promote frequent A3B-induced mutations.

**Table 1:** Gene ontology (GO) of proteins with increased binding to A3B by MNNG or Act D

MNNG					
**#term ID**	**Term description**	**Observed gene count**	**Background gene count**	**Strength**	**False discovery rate**
GO:0030623	U5 snRNA binding	2	2	2.33	0.0233
GO:0004013	Adenosylhomocysteinase activity	2	3	2.15	0.0337
GO:0097157	pre-mRNA intronic binding	3	13	1.69	0.0135
GO:0043024	Ribosomal small subunit binding	3	17	1.58	0.0206
GO:0036002	pre-mRNA binding	6	35	1.56	1.66E-05
GO:0003724	RNA helicase activity	9	76	1.4	1.48E-07
GO:0035925	mRNA 3-UTR AU-rich region binding	3	26	1.39	0.0446
GO:0017069	snRNA binding	4	47	1.26	0.0198
GO:0003730	mRNA 3-UTR binding	7	102	1.17	0.00029
GO:0003725	Double-stranded RNA binding	5	76	1.15	0.0103
GO:0043021	Ribonucleoprotein complex binding	8	152	1.05	0.00029
GO:0003729	mRNA binding	15	326	0.99	5.02E-08
GO:0003697	Single-stranded DNA binding	5	120	0.95	0.0422
GO:0140098	Catalytic activity, acting on RNA	14	380	0.9	2.06E-06
GO:0003723	RNA binding	60	1672	0.89	2.25E-37
GO:0016887	ATP hydrolysis activity	10	333	0.81	0.0014
GO:0045296	Cadherin binding	10	334	0.81	0.0014
GO:0140657	ATP-dependent activity	11	543	0.64	0.0135
GO:0050839	Cell adhesion molecule binding	11	560	0.62	0.0152
GO:0017111	Nucleoside-triphosphatase activity	11	650	0.56	0.0366
GO:0003676	Nucleic acid binding	63	4003	0.53	9.35E-20
GO:1901363	Heterocyclic compound binding	71	5977	0.41	5.71E-17
GO:0030554	Adenyl nucleotide binding	19	1566	0.41	0.0206
GO:0005524	ATP binding	18	1491	0.41	0.0299
GO:0097159	Organic cyclic compound binding	71	6050	0.4	9.04E-17
GO:0097367	Carbohydrate derivative binding	26	2278	0.39	0.0033
GO:0017076	Purine nucleotide binding	22	1917	0.39	0.0143
GO:0035639	Purine ribonucleoside triphosphate binding	21	1834	0.39	0.0198
GO:0032555	Purine ribonucleotide binding	21	1903	0.37	0.0284
GO:0005488	Binding	86	12,838	0.16	1.32E-07

Act D					
GO:0030623	U5 snRNA binding	2	2	2.37	0.0154
GO:0050733	RS domain binding	2	5	1.97	0.0479
GO:0030515	snoRNA binding	5	31	1.58	0.00012
GO:0003724	RNA helicase activity	8	76	1.39	1.93E-06
GO:0015035	Protein-disulfide reductase activity	4	39	1.38	0.0054
GO:0017069	snRNA binding	4	47	1.3	0.0097
GO:0004386	Helicase activity	10	149	1.2	1.38E-06
GO:0019843	rRNA binding	4	67	1.15	0.0315
GO:0051087	Chaperone binding	5	108	1.04	0.0169
GO:0016887	ATP hydrolysis activity	13	333	0.96	1.93E-06
GO:0003723	RNA binding	58	1672	0.91	3.11E-38
GO:0140098	Catalytic activity, acting on RNA	12	380	0.87	3.19E-05
GO:0140657	ATP-dependent activity	14	543	0.78	3.06E-05
GO:0017111	Nucleoside-triphosphatase activity	16	650	0.76	6.56E-06
GO:0140640	Catalytic activity, acting on a nucleic acid	14	575	0.76	5.30E-05
GO:0016462	Pyrophosphatase activity	17	705	0.75	3.84E-06
GO:0003676	Nucleic acid binding	62	4003	0.56	2.92E-22
GO:0005524	ATP binding	20	1491	0.5	0.00081
GO:0035639	Purine ribonucleoside triphosphate binding	24	1834	0.49	0.00013
GO:1901265	Nucleoside phosphate binding	26	2169	0.45	0.00019
GO:0032555	Purine ribonucleotide binding	23	1903	0.45	0.00076
GO:0000166	Nucleotide binding	25	2168	0.43	0.00063
GO:1901363	Heterocyclic compound binding	66	5977	0.41	2.10E-16
GO:0097159	Organic cyclic compound binding	66	6050	0.41	3.18E-16
GO:0016787	Hydrolase activity	26	2347	0.41	0.00074
GO:0036094	Small molecule binding	26	2507	0.39	0.0018
GO:0097367	Carbohydrate derivative binding	24	2278	0.39	0.0034
GO:0005488	Binding	78	12,838	0.15	1.93E-06

Proteins interacting with A3B after DNA damage by Act D (1 µM) or MNNG (50 µM) were analyzed by IP-MS DIA analysis using A3B-AcGFP-expressing cells and AcGFP-expressing cells as control. The threshold value was set at five-fold of the protein quantification value in AcGFP-expressing cells. Of these, 92 proteins (MNNG, panel A) and 84 proteins (Act D, panel B) with more than a 10-fold increase were categorized by molecular function using GO via STRING ver. 12.

**Table 2:** Localization of detected RNA helicases

Gene name	Location
DDX17	Nuclear speckles
DDX3X	Membrane, intracellular
EIF4A1	Cytosol
DHX9	Nucleoplasm and nucleoli
DDX5	Nucleoplasm and nucleoli
UPF1	Nucleoplasm and cytosol
EIF4A3	Nucleoplasm
DDX21	Nucleoli rim, mitotic chromosome, and nucleoplasm
DHX15	Nuclear speckles
DHX30	Mitochondria and cytosol
DDX47	Nucleoli
DDX46	Nuclear speckles and nucleoli fibrillar center
SNRNP200	Nucleoplasm
DDX27	Nucleoli
DDX1	Nucleoplasm and cytosol
DDX18	Nucleoli, nucleoli rim, and mitotic chromosome
G3BP1	Cytosol
MTREX	Nucleoplasm
DDX56	Nucleoli and mitotic chromosome
DDX23	Nucleoplasm and nucleoli
DHX8	Nucleoplasm and nuclear bodies
DDX6	Cytoplasmic bodies and cytosol
DHX57	Intermediate filaments, microtubules, nucleoli, and cytosol

These localizations were obtained from the HUMAN PROTEIN ATLAS web site (https://www.proteinatlas.org/).

## Discussion

A3B expression correlates with the enrichment of A3B-derived mutations in cancer cells, and A3B-induced mutations are introduced into ssDNA during replication and transcription [21]. Another mechanism for A3B-mediated mutagenesis is that A3B is induced or activated by DNA damage and subsequently generates delayed secondary DSBs [47]. APOBEC3 induces cluster mutations such as kataegis and omikli and also causes an accumulation of mutations, called mutation showers, in the vicinity of DNA breaks [19]. Although changes in the nuclear localization of A3B by viruses have been reported [48], it remains unclear whether DNA damage or other factors alter the nuclear localization of A3B and how A3B accumulates at the site of DNA damage. In this study, we showed that A3B was associated with an increase in long deletions after irradiation and speculated that the long deletions were caused by radiation- and A3B-induced DNA damage occurring simultaneously in the vicinity, complicating DNA damage [20]. The purpose of this study was to test the hypothesis that DNA damage triggers A3B to bind to ssDNA in the R-loop generated in the vicinity of DNA damage. Therefore, we induced sustained DNA damage with the drug and observed any changes in the nuclear localization of A3B.

The RNA-binding protein hnRNPC1/2, located in the nuclear matrix, exhibits RNA-independent binding to A3B [18]. A3B and hnRNPC1/2 show nearly identical nuclear localization during both interphase and mitosis (Figure 1 and S-Figure 8) [49]. RNase A treatment of live cells eliminates hnRNPC1/2 from the nucleus [50]. While permeabilization of living cells preserves the nucleus, nucleolus, and microtubules, subsequent RNase A treatment disrupts the nucleolus and several nuclear structures, including RNA and components of the nuclear matrix [51–55]. Consistent with this, RNase A treatment led to the removal of both hnRNPC1/2 and A3B from the nucleus, accompanied by loss of the nuclear Nucleolus Bright Red signal (S-Figure 1). These results indicate that RNA or RNA-containing nuclear structures are essential for retaining A3B in the nuclear matrix. However, the binding of A3B to the nuclear matrix and other nuclear structures is likely attributable to proteins and RNAs other than hnRNPC1/2. This hypothesis is supported by the results of co-immunoprecipitation experiments using VHH antibodies, which demonstrated a low level of binding of hnRNPC1/2 to A3B (data not shown) and the distribution of both proteins in the nuclear matrix in the confocal microscope images does not perfectly match (Figure 1).

Live-cell imaging was performed to examine the localization changes of A3B after DNA damage. Upon addition of MNNG, A3B dispersed in the nucleus, with a portion entering the nucleolus region (S-Figure 9). hnRNPC1/2 did not migrate to the nucleolus during the accumulation of A3B in the nucleolus by MNNG (S-Figure 10). This indicates that A3B migrated from the nuclear matrix to the nucleolus rather than the nuclear matrix invading the nucleolus. After 3 h of MNNG treatment, a portion of A3B migrated to the DNase I-extracted fraction (S-Figure 11). A3B accumulation in the nucleolus was abolished by DNase I, but not by RNase A treatment (Figure 5B). These results indicate that MNNG causes a portion of A3B to migrate from the nuclear matrix to the nucleolus and bind to DNA. Although different from the pattern of A3B accumulation by MNNG, TOPO I inhibition also accumulated A3B in the nucleolus (Figure 2). In this study, we focused on A3B localized in the nucleolus; however, residual A3B may remain in the nucleoplasm even after RNase A treatment. For example, in ETO-treated cells, A3B appeared to persist in the nucleoplasm after RNase A treatment (Figure 4A). Although not shown here, doxorubicin-treated cells showed a point-like accumulation of A3B in the nucleoplasm. Therefore, investigating the mechanism of action and effects of this differential accumulation in future studies would be beneficial. Furthermore, the nuclear matrix is involved in the DDR. For example, Rad51 binds to the nuclear matrix after DNA damage [56]. Consequently, an analysis of A3B accumulation in the nucleoplasm is expected to further our understanding of nuclear matrix functions.

We attempted to elucidate the mechanisms underlying A3B accumulation, but this proved challenging due to the nucleolar localization of A3B. Detecting DNA strand breaks within the nucleolus is inherently difficult. DSBs are rarely visualized in this compartment, likely due to the low nucleosome occupancy of ribosomal DNA (rDNA) and the corresponding scarcity of γ-H2AX signals [57]. Although PARP-1 and XRCC1, indicators of SSB, are located in the nucleolus, they migrate to the nucleoplasm when SSB occurs [31]. Therefore, we cannot rule out the possibility that A3B binds to DSB or SSB sites present in the nucleolus.

R-loop visualization in the nucleolus is also technically limited. Most studies use S9.6 antibody-based immunostaining to detect DNA/RNA hybrids and report strong S9.6 signals in both the cytoplasm and nucleolus. The presence of R-loops in the nucleolus is supported by studies showing their accumulation during RNAP I transcription and formation by RNAP II within the nucleolus [58,59]. However, the persistent, RNase H-insensitive S9.6 signal in the nucleolus is thought to reflect the abundance of ribosomal RNA rather than true DNA/RNA hybrids [60]. Although methanol fixation provides superior immunostaining with the S9.6 antibody compared with PFA [61], PFA fixation was performed for comparison. This is because the S9.6 signal in the nucleolus that can be detected with methanol fixation is not believed to originate from DNA/RNA hybrids. However, no favorable results were obtained. Recently, RNase H1 mutants that specifically bind R-loops have been used to visualize their subcellular localization in living cells [58]. However, given the high basal levels of both wildtype and mutant RNase H1 in the nucleolus, it is difficult to distinguish genuine R-loop binding events from background signal. Currently, the most reliable approach to infer R-loop presence in the nucleolus involves removing single- and double-stranded RNA entirely, followed by S9.6 immunostaining or monitoring changes after RNase H treatment, which specifically degrades DNA/RNA hybrids. In this study, we used RNase H treatment for comparison. Both CPT and Act D are known to promote R-loop formation [62–64]. In particular, RNAP I inhibition by these drugs has been shown to cause accumulation of S9.6 signal and RNase H1 at the nucleolar periphery, indicating R-loop formation at these sites [58]. This observation is consistent with our findings, where A3B accumulation at the nucleolar periphery was abolished by RNase H (Figure 3A). However, since the R-loop of the nucleolus cannot be directly visualized with the S9.6 antibody, another tool would be needed to study its colocalization with A3B.

In this study, the pattern of A3B accumulation in the nucleolus varied slightly depending on the drug used. Act D and CPT, which induce DSBs in the nucleoplasm, also cause TOPO I to accumulate at the nucleolar periphery [65]. Inhibition of TOPO I, i.e., stabilization of TOPO I/DNA covalent complexes, was considered a factor in the accumulation of A3B. In MNNG-treated cells, A3B accumulated not only peripherally within the nucleolus but also internally. Given that MNNGs generate AP sites that contribute to R-loops, we investigated whether MNNGs induce the appearance of AP sites in the nucleolus. AP sites in DNA can be quantitatively detected in vitro using ARPs [66–68]. However, this technique has rarely been used to study the colocalization of AP sites with other proteins using cell staining. Successful ARP staining of AP sites in situ relies heavily on high-resolution confocal microscopy. Moreover, since AP sites can also exist in RNA [69], removal of background RNA is essential. Therefore, membrane permeabilization and RNase A treatment were key steps in the accurate quantification of DNA-derived AP sites. Generally, damaged bases resulting from the action of DNA-alkylating agents are removed by DNA glycosylases, leading to the formation of AP sites, which are subsequently removed using apurinic/apyrimidinic endonuclease 1 (APE1). Excessive DNA damage is also removed by APE2 [70]. APE1 and APE2 are mainly found in the nucleus; however, live-cell imaging has revealed that APE1 is more abundant in the nucleolus and euchromatin sites [71]. Although the DNA glycosylases that generate AP sites are primarily located in the nucleoplasm, some studies have reported their occurrence in the nucleolus [72,73]. Therefore, the observed increase in MNNG-induced ARP staining in the nucleolus appeared to be a reasonable consequence of the DDR, given the presence of APE1 in the nucleolus. However, DDR differs between the nucleolus and the remainder of the nucleus [38]. Although the significance of the nucleolar DDR (n-DDR) in maintaining genome stability has been established, numerous aspects of rDNA processing, nucleolus segregation, and nucleolus cap formation remain unclear. Moreover, it remains unclear whether AP sites are formed in the nucleolus, or whether AP sites generated in the nucleoplasm accumulate in the nucleolus.

The IP-LC/MS/MS DIA analysis employing A3B as bait was designed for the purpose of detecting alterations in A3B-interacting proteins in instances where A3B undergoes changes in its subcellular location, specifically transitioning from the nuclear matrix to the nucleolus. The analysis is currently ongoing and focuses on the release of A3B from the nuclear matrix, the augmentation of DNA repair proteins, and the elucidation of its mechanism of action. Proteomic analysis of A3B-interacting proteins revealed increased interactions with nuclear RNA helicases following Act D or MNNG treatment (Figure 6A, Tables 1 and 2). Several RNA helicases have been identified as R-loop binding proteins [74]. These RNA helicases are believed to play a role in R-loop formation and resolution, with DDX1, DDX17, and DHX9 as illustrative examples [75]. Consistent with the results of MS DIA analysis, following treatment with Act D or MNNG, DDX17 relocated to the sites of A3B accumulation in the nucleolus rim and interior (S-Figure 12). Furthermore, colocalization of DDX17 and A3B was also observed following CPT treatment. DDX21 is highly expressed in the nucleolar rim, whereas DDX46 is highly expressed in the FC or nuclear speckles [76,77]. However, DDX21 is excluded from the nucleolus by Act D treatment, so the interaction with A3B would have occurred in the nucleoplasm [78]. DDX21 is reportedly involved in the modification of N6-methyladenosine in the R-loop, while DDX46 is involved in the demethylation of N6-methyladenosine [79,80]. Additionally, the levels of ILF3, a component of the NFTA (ILF2-ILF3 complex) that binds to DHX9, were markedly increased, similar to those of DNA-PKcs (PRKDC), which bind to ILF3 (S-Figure 13) [81]. PRKDC interacts with XRCC5 (Ku80) and XRCC6 (Ku70), which bind to the ends of DSBs. However, the level of interaction between XRCC5/XRCC6 did not increase significantly, indicating that the A3B complex may contain a limited number of DSBs. This result was consistent with the immunostaining results, which demonstrated that A3B did not colocalize effectively with DSBs (Figure 2B). Furthermore, XRCC1, which binds to SSB, was not detected. A notable increase in proteins with functional RNA-binding capacity and involved in RNA splicing was also observed (Tables 1 and 2). Certain RNAs are believed to mediate phase separation at DNA damage sites and assist in recruiting DNA damage repair factors. For instance, various DSB damage repair factors and RNAs, including pre-rRNA-related proteins and snoRNAs, colocalize in the XY body during pre-meiosis [82]. These results suggest that after DNA damage, A3B translocates to RNA helicase-rich compartments and proteins involved in RNA splicing, e.g., nuclear speckle.

In our preliminary experiments, many drugs, including ETO and cisplatin, induced DNA damage but did not accumulate A3B in the nucleolus. To clarify why A3B migrates to the nucleolus and whether A3B actually binds to the R-loop in the nucleolus, it will be necessary to elucidate the effects of drugs on the nucleolus as well as n-DDR’s mechanism of action. Moreover, it will be necessary to visualize the R-loop in the nucleolus.

This study demonstrated that DNA damage resulting from TOPO I inhibition or DNA alkylation induced RNase H-sensitive accumulation of A3B at the nucleolus but not at DSB sites, independent of S9.6 antibody staining. This accumulation of A3B in response to DNA damage may increase local DNA damage and cause mutation showers. The accumulation of A3B-mediated mutations in cancer cells contributes to the acquisition of diverse traits, including drug resistance. Furthermore, DNA binding within the A3B nucleosome suggests that A3B influences carcinogenesis and cancer development through damage or mutation of ribosomal DNA (rDNA). The effects of anticancer drugs such as TOPO I inhibitors and DNA alkylating agents on the nucleolus are interpreted as side effects in cancer treatment. However, in cancer cells expressing A3B, it is speculated that these drugs exert a more substantial effect by inhibiting cellular function through A3B accumulation in the nucleolus and its DNA binding. The results of this study provide new insights into A3B’s role in cancer heterogeneity and highlight the need for further investigation into the nucleolar DDR, an area that remains largely unexplored.

## Materials and methods

### Cell culture

HepG2 (human hepatocellular carcinoma) cell lines were obtained from the Cell Resource Center for Biomedical Research, Institute of Development, Aging, and Cancer, Tohoku University, Sendai, Japan. APOBEC3B-AcGFP vectors were constructed by inserting APOBEC3B-ORF and a linker into pAcGFP1-N1 plasmids (Takara Bio Inc., Shiga, Japan). This plasmid was transfected into HepG2 cells using FuGENE-HD (Promega Corp., WI, U.S.A.), and stably expressing APOBEC3B-AcGFP (A3B-AcGFP) cell lines were selected using G418 (final concentration at 1 mg/ml) (Nacalai Tesque, Inc., Kyoto, Japan). APOBEC3B-knockout HepG2 (A3B KO) cells were generated using target-specific CRISPR/Cas9 knockout plasmids (Santa Cruz Biotechnology, Inc., CA, U.S.A.). Subsequently, the cells were cultured in RPMI1640 (Nacalai Tesque, Inc.) containing 5% fetal calf serum (Biological Industries, Kibbutz Beit Haemek, Israel) in a humidified 5% CO_2_ atmosphere at 37°C.

### Reagents

Act D, MgCl_2_, EGTA, Triton-X-100, and Tween 20 were purchased from Nakalai Tesque, Inc. Aphidicolin was procured from Sigma-Aldrich Japan LLC. (Tokyo, Japan), and PFA, MNNG, CPT, ETO, MPA, hydroxycarbamide, and 6-TG were obtained from FUJIFILM Wako Pure Chemical Corp., (Osaka, Japan).

### Immunofluorescence and image analysis

For hnRNPC1/2 staining, A3B-AcGFP-expressing HepG2 cells were plated on CellCarrier-96 Ultra Microplates (Revvity Inc., MA, U.S.A.). Cells were fixed with 4% PFA in PBS for 10 min at 20–25°C (room temperature, RT). All experimental manipulations described hereafter were performed entirely at RT unless otherwise noted. Cells were washed/permeabilized with 0.5% Triton X100 in PBS for 5 min, blocked with Blocking One Histo (Nacalai Tesque, Inc.) for 1 h, stained with anti-hnRNPC1/2 antibody- Alexa Fluor 647 (1/1,000 dilution, ab208765, Abcam plc., Cambridge, UK) for 1 h. The stained cells were washed with 0.1% Tween 20 in PBS (PBS-T), stained with 1 µmol/l of Nucleolus Bright Red (Dojindo Laboratories Co., Ltd., Kumamoto, Japan) for 10 min, and HOECHST 33342 (HOECHST) (Thermo Fisher Scientific, Inc, MA, U.S.A.) for 10 min.

For nucleolus staining, A3B-AcGFP-expressing HepG2 cells were plated on CellCarrier-96 Ultra Microplates and incubated in a humidified 5% CO_2_ atmosphere at 37°C overnight. Cells were treated with Act D (1  µM), CPT (1  µM), ETO (50  µM), or MNNG (50  µM) for 3  h, or alternatively with MPA (10  µM), Act D (10  nM), hydroxycarbamide (0.5  mM), 6-TG (20  µg/ml), or aphidicolin (30  µM) for 24  h under the same incubation conditions. The cells were then washed with PBS, fixed with 4% PFA in PBS for 10 min, permeabilized with 0.5% Triton X100 in PBS for 5 min, and blocked with Blocking One Histo for 1 h. Subsequently, the cells were stained with Nucleolus Bright Red (1 µmol/l) for 10 min, washed with PBS-T, and stained with HOECHST for 10 min.

For γ-H2A.X staining, A3B-AcGFP-expressing HepG2 cells were plated on CellCarrier-96 Ultra Microplates and incubated in a humidified 5% CO_2_ atmosphere at 37°C overnight. The cells were incubated with Act D (1 µM), CPT (1 or 10 µM), ETO (50 µM), or MNNG (50 µM) for 3 h in a humidified 5% CO_2_ atmosphere at 37°C. Subsequently, the cells were washed with PBS, fixed with 4% PFA in PBS for 10 min, permeabilized with 0.5% Triton-X-100 for 5 min, blocked with Blocking One Histo for 1 h, and incubated with anti-γ-H2A.X antibody - Alexa Fluor 594 (1/1,000 dilution, ab206898, Abcam plc.) for 1 h. Finally, the cells were washed with PBS-T and stained with Nucleolus Bright Red (1 µmol/l) for 10 min, and HOECHST for 10 min.

For S9.6 staining, APOBEC3B-AcGFP-expressing HepG2 cells were plated on CellCarrier-96 Ultra Microplates and incubated in a humidified 5% CO_2_ atmosphere at 37°C overnight. The cells were incubated with Act D (1 µM), CPT (1 µM), or MNNG (50 µM) for 3 h in a humidified 5% CO_2_ atmosphere at 37°C, washed with PBS, and permeabilized with 0.1% Triton-X 100 for 1 min. Subsequently, the cells were treated with RNase A (10  µg/ml; NIPPON GENE, Tokyo, Japan) in PBS containing a protease inhibitor cocktail (Nacalai Tesque, Inc.); RNase H (6 U/well; Takara Bio) in PBS supplemented with 4  mM MgCl₂ and protease inhibitor cocktail; or DNase I (1 U/well; NIPPON GENE) in DNase I buffer (40  mM Tris-HCl, 10  mM NaCl, 6  mM MgCl₂, 1  mM CaCl₂, protease inhibitor cocktail; pH 7.9). Cells were then fixed with cold methanol (MeOH, −20°C) for 10 min at 4°C. The cells were blocked with Blocking One Histo for 1 h, stained with a primary anti-DNA/RNA hybrid antibody (S9.6, rabbit IgG, 1/1000 dilution, ab01137-23.0) (Absolute Antibody Ltd., OH, U.S.A.) for 1 h, washed with PBS-T, and incubated with a secondary anti-rabbit IgG antibody conjugated with Ax594 (1/2,000 dilution, ab 150080) for 1 h. Finally, the cells were washed with PBS-T and stained with HOECHST for 10 min.

All cell images were captured using an LSM 900 with Airyscan 2 detector in super resolution (SR) mode (Microscope: Axio Observer. Z1 / 7; Objective: Plan-Apochromat 63×/1.40 Oil DIC M27). Images were acquired using ZEN software (Carl Zeiss MicroImaging, Inc., Germany) with a field size of 78.01 µm × 78.01 µm at a resolution of 2,210 × 2210 pixels and 16-bit depth. Subset images (600 × 600 pixels) centered on individual cells were generated. Image post-processing was limited to setting a uniform fluorescence threshold across all images to match control cells (approximately black: 2.0%; white: 0.1%). The gamma value was set to 1.0, and no further image manipulation was applied.

### ARP staining

A3B KO HepG2 cells (A3B KO) or APOBEC3B-AcGFP-expressing HepG2 cells were plated on CellCarrier-96 Ultra Microplates and incubated in a humidified 5% CO_2_ atmosphere at 37°C overnight. The cells were incubated with MNNG (50 µM), Act D (1 µM), or ETO (50 µM) for 3 h in a humidified 5% CO_2_ atmosphere at 37°C. The cells were permeabilized with 0.1% Triton-X 100 for 1 min and treated with or without RNase A (10 µg/ml) in PBS containing a protease inhibitor cocktail for 30 min. The cells were then washed with PBS and fixed with MeOH for 10 min at 4°C, blocked using an Endogenous Avidin-Biotin Blocking Kit (Nichirei Bioscience, Inc., Tokyo, Japan), and incubated with ARP (10 µg/ml) (A305, Dojindo Laboratories Co., Ltd.) for 1 h. After washing with PBS, the cells were blocked with Blocking One Histo for 1 h and stained with Streptavidin- Alexa Fluor 647 (ab272190, Abcam plc.) for 1 h, washed with PBS-T, stained with Nucleolus Bright Red (1 µmol/l) for 10 min, and then with HOECHST for 10 min. All cell images were captured using an LSM 900 with Airyscan 2, and data processing was performed using ZEN software. A magnified subset image (ZOOM; 130 × 129 pixels) in Figure 5A was extracted from a 4.59 µm × 4.55 µm region of the original image. For visualization, fluorescence intensity thresholds were adjusted: APR signal thresholds were set from 869 to 566 (white) and 10 to 30 (black); A3B signal thresholds were set from 2941 to 1033 (white) and 4 to 58 (black).

### Immunoprecipitation and DIA analysis

APOBEC3B-AcGFP-expressing HepG2 or AcGFP-expressing HepG2 cells plated on 10 cm dishes were stimulated with Act D (1 µM) or MNNG (50 µM) for 3 h. The cells were washed three times with PBS and lysed with RIPA buffer (Nacalai Tesque, Inc.) containing a protease inhibitor cocktail. The protein amounts in the cell lysates were measured using a BCA protein assay (Sigma-Aldrich, MO, U.S.A.) by mixing 0.1 ml of the cell lysate (5 mg/ml) with 4 µl of anti-GFP, GFP-Trap Magnetic Agarose beads (Proteintech Group, Inc., IL, U.S.A.) and centrifuging for 1 h at 4°C. The beads were washed three times with TBS-T, suspended in 100 µl of 50 mM Tris-HCl pH 8.0, added to 500 ng of trypsin/Lys-C mix, and incubated at 37°C overnight. The supernatant was mixed with TCEP (final conc. 20 mM) and incubated at 80°C for 10 min. The samples were mixed with iodoacetamide (final conc. 30 mM), incubated for 30 min at RT. Subsequently, 5% TFA was added. The samples were subjected to reverse-phase liquid chromatography (GL-Tip SDB, GL Sciences, Inc., Tokyo, Japan), followed by LC-MS analysis. The peptides were directly injected onto a 75 μm × 20 cm PicoFrit emitter (CAT# PF360-75-8-N-5, New Objective, Woburn, MA, U.S.A.) packed in-house with C18 core−shell particles (CAT# 51,227 (disassembled the column and got the particles), CAPCELL CORE MP 2.7 μm, 160 Å material; Osaka Soda, Osaka, Japan) at 50°C and then separated with a 60 min gradient at a flow rate of 200 nl/ min using an UltiMate 3000 RSLCnano LC system (Thermo Fisher Scientific). Peptides eluting from the column were analyzed on a Q Exactive HF-X (Thermo Fisher Scientific) for overlapping window DIA-MS. The MS1 spectra were collected in the range of m/z 495–745 at 30,000 resolution to set an automatic gain control (AGC) target of 3 × 10^6^. MS2 spectra were collected in the range of more than 200 m/z at 30,000 resolution to set an AGC of 3 × 10^6^, a maximum injection time of ‘auto’, stepped normalized collision energies of 23% and isolation window of 4.0 m/z. The predicted spectrum library was created using DIA-NN 1.8.1 under the following conditions. Human UniProtKB/SwissProt database (proteome ID UP000005640, 20,588 entries, downloaded on November 26, 2021), FASTA digest for library free search, trypsin as the digestive enzyme (allowing up to one cleavage defect), and cysteine carbamoylation. Using the predicted spectral library, protein and peptide identification and quantification were performed using DIA-NN 1.81 under the following conditions: Mass accuracy, 10 ppm; Fragment Tolerance, 10 ppm; Precursor FDR, 1% or less; Protein FDR, 1% or less; Unrelated runs, On; Use isotopologues, On; MBR, On; Heuristic protein inference, On; No shared spectra, On; Protein inference, Genes; Neural network classifies, Single-pass mode; Quantification strategy: Robust LC (high precision). Mass spectrometry and sample preparation were performed by Kazusa DNA Research. Inst. software (Chiba, Japan). The accession numbers are PXD058962 for ProteomeXchange and JPST003516 for jPOST [83].

A total of 5574 protein data points were obtained from APOBEC3B-AcGFP-expressing HepG2 cells, 2174 from AcGFP-expressing HepG2 cells, 3329 from Act D-treated APOBEC3B-AcGFP-expressing HepG2 cells, and 2412 from MNNG-treated APOBEC3B-AcGFP-expressing HepG2 cells. The threshold value was set at five-fold that of the protein quantification value in AcGFP-expressing cells. Only proteins with quantitation values above the threshold were analyzed in APOBEC3B-AcGFP-, Act D-treated APOBEC3B-AcGFP-, and MNNG-treated APOBEC3B-AcGFP-expressing HepG2 cells. A total of 92 proteins in MNNG-treated cells and 84 proteins in Act D-treated cells showed more than a 10-fold increase in quantitative values relative to control cells. These proteins were classified according to molecular function using GO analysis via STRING ver. 12.

### Statistical analysis

Statistical analysis of S9.6 staining intensity in the nucleus and A3B staining intensity in the nucleolus for each drug treatment was performed using Tukey’s multiple comparison test in GraphPad Prism 8 (GraphPad Software Inc., MA, U.S.A.). Multiple testing for the ARP staining intensity in the nucleus or the A3B intensity in the nucleolus was analyzed using Dunnett’s test in GraphPad Prism 8. Statistical significance was defined as *P<0.05 and **P<0.01.

## Supplemental methods

### Extraction of hnRNPC1/2 and A3B from the nucleus by RNase A or DNase I treatment

A3B-AcGFP-expressing HepG2 cells were plated on CellCarrier-96 Ultra Microplates and incubated in a humidified 5% CO_2_ atmosphere at 37°C overnight. Cells were then permeabilized with 0.1% Triton-X-100 in PBS for 1 min, followed by treatment with either RNase A (10 µg/ml) in PBS supplemented with protease inhibitor cocktail or DNase I (1 U/well) in DNase I buffer (40 mM Tris-HCl, 10 mM NaCl, 6 mM MgCl₂, 1 mM CaCl₂, protease inhibitor cocktail, pH 7.9) for 30 min at RT. After enzymatic treatments, cells were fixed with cold methanol (–20°C) for 10 min at 4°C. The cells were blocked with Blocking One Histo for 1 h, then stained with anti-hnRNPC1/2 antibody-Alexa Fluor 647 (1/1,000 dilution, ab208765) for 1 h. The stained cells were washed with PBS-T, then counter-stained with HOECHST and/or Nucleolus Bright Red (1 µmol/l) for 10 min. Nuclear regions were identified based on HOECHST staining, and fluorescence intensity histograms for hnRNPC1/2 and A3B signals were generated on a per-pixel basis within these regions.

### Act D, MNNG, and CPT treatment in living cells expressing A3B-AcGFP

HepG2 cells were plated on CellCarrier-96 Ultra Microplates and incubated in a humidified 5% CO_2_ atmosphere at 37°C overnight. Act D (1 µM), MNNG (50 µM), or CPT (10 µM) was added to the cells, and the cells were imaged 15 min later.

### S9.6 staining in RNase A treatment after methanol fixation and before PFA fixation

A3B-AcGFP-expressing HepG2 cells were plated on CellCarrier-96 Ultra Microplates and incubated in a humidified 5% CO_2_ atmosphere at 37°C overnight. Cells were treated with Act D (1 µM), CPT (1 µM), or MNNG (50 µM) for 3 h at 37°C. After treatment, cells were washed with PBS and either fixed in cold methanol (–20°C) for 10 min at 4°C (for S-Figure 3A) or fixed with 4% paraformaldehyde (PFA) for 10 min followed by permeabilization with 0.1% Triton-X-100 for 1 min (for S-Figure 5A). Subsequently, cells were incubated with RNase A (10 µg/ml) in PBS containing protease inhibitors or RNase H (6 U/well) in PBS supplemented with 4 mM MgCl₂ and protease inhibitors for 30 min prior to immunostaining. The cells were blocked with Blocking One Histo for 1 h, stained with a primary anti-DNA/RNA hybrid antibody (S9.6, rabbit IgG, ab01137-23.0) for 1 h, washed with PBS-T, and incubated with a secondary anti-rabbit IgG antibody conjugated with Ax594 (1/2,000, ab 150080) for 1 h. Finally, the cells were washed with PBS-T and stained with HOECHST for 10 min.

### S9.6 staining in CPT-treated cells

A3B-AcGFP-expressing HepG2 cells were plated on CellCarrier-96 Ultra Microplates and incubated in a humidified 5% CO_2_ atmosphere at 37°C overnight. The cells were incubated with CPT (1 µM) for 5 or 20 min. Subsequently, the cells were permeabilized with 0.1% Triton X-100 for 1 min and treated with RNase A (10 µg/ml) in PBS containing protease inhibitor cocktail. The cells were blocked with Blocking One Histo for 1 h, stained with a primary anti-DNA/RNA hybrid antibody (S9.6, rabbit IgG, ab01137-23.0) for 1 h, washed with PBS-T, and incubated with a secondary anti-rabbit IgG antibody conjugated with Ax594 (1/2,000, ab 150080) for 1 h. Finally, the cells were washed with PBS-T and stained with HOECHST for 10 min. Nuclear regions were identified based on HOECHST staining, and S9.6 fluorescence intensity within the nucleolus was quantified (*n* = 4).

### ARP staining for extracted DNA

HepG2 cells were plated on 10 cm dishes and incubated with/without MNNG for 3 h. DNA was extracted from the cells using the Wizard Genomic DNA Purification kit, following the protocols provided with the reagent. The extracted DNA was air-dried on CellCarrier-96 Ultra Microplates, then fixed and blocked with Blocking One Histo reagent for 1 h. Endogenous biotin was blocked using an Avidin-Biotin Blocking Kit according to the manufacturer’s instructions. Samples were then incubated with ARP (10 µg/ml) for 1 h. After washing with PBS, the DNA was blocked with Blocking One Histo for 1 h and stained with Streptavidin-Alexa Fluor 647 (ab272190) for 1 h, washed with PBS-T, then stained with HOECHST for 10 min.

Images were captured using an LSM 900 with an Airyscan 2 detector in SR mode (Microscope: Axio Observer. Z1 / 7; Objective: Plan-Apochromat 63×/1.40 Oil DIC M27). The images were acquired using ZEN software in a 77.16 µm × 77.16 µm area at 2,186 × 2186 pixels, 16-bit depth. Fluorescence intensity profiles in ARP and HOECHST were acquired using ZEN software.

### ARP staining for RNase A-treated cells

A3B-AcGFP-expressing HepG2 cells were plated on CellCarrier-96 Ultra Microplates and incubated in a humidified 5% CO_2_ atmosphere at 37°C overnight. The cells were incubated with MNNG (50 µM) for 3 h in a humidified 5% CO_2_ atmosphere at 37°C. RNA treatment, fixation, and ARP staining of cells were performed as described in the Materials and Methods. Nuclear regions were identified via HOECHST staining, and fluorescence intensity histograms of ARP staining per pixel within the nuclei were generated.

### hnRNPC1/2 staining

hnRNPC1/2 staining was conducted in the same way as described in the Materials and Methods.

### DDX17 staining

A3B-AcGFP-expressing HepG2 cells were plated on CellCarrier-96 Ultra Microplates and incubated in a humidified 5% CO_2_ atmosphere at 37°C overnight. The cells were incubated with Act D (1 µM), MNNG (50 µM), or CPT (10 µM) for 3 h in a humidified 5% CO_2_ atmosphere at 37°C. Subsequently, the cells were washed with PBS, fixed with 4% PFA in PBS for 10 min, permeabilized with 0.5% Triton-X-100 for 5 min, blocked with Blocking One Histo for 1 h, stained with a primary anti-DDX17 antibody (rabbit IgG, 1/1000 dilution, 19910–1-AP) (Proteintech Group, Inc., IL, U.S.A.) for 1 h, washed with PBS-T, and incubated with a secondary anti-rabbit IgG antibody conjugated with Ax594 (1/2,000 dilution, ab 150080) for 1 h. Finally, the cells were washed with PBS-T and stained with HOECHST for 10 min.

### MNNG and ETO treatment in living cells

A3B-AcGFP-expressing HepG2 cells were plated on CellCarrier-96 Ultra Microplates and incubated in a humidified 5% CO_2_ atmosphere at 37°C overnight. Cells were stained with HOECHST for 20 min, followed by three washes with PBS. MNNG (50 µM) or etoposide (ETO, 50 µM) was added directly on the microscope stage, and live-cell imaging was performed at 5, 10, 30, and 60 min post-treatment.

### Image analysis

All cell images except 3D z-stack images were captured using an LSM 900 with an Airyscan 2 detector in SR mode (Microscope: Axio Observer. Z1 / 7; Objective: Plan-Apochromat 63×/1.40 Oil DIC M27). All images were acquired using ZEN software.

### 3D z-stack imaging

3D z-stack cell images were captured using an LSM 900 with an Airyscan 2 detector in SR mode (Microscope: Axio Observer. Z1 / 7; Objective: Plan-Apochromat 63×/1.40 Oil DIC M27). All images were acquired using ZEN software over a 78.01 µm × 78.01 µm field at 2,210 × 2210 pixels with 16-bit depth. Z-stacks comprised 40 slices at 0.13 µm intervals.

### Nuclear fractionation

A3B-AcGFP-expressing HepG2 cells were plated on 10 cm dishes and incubated in a humidified 5% CO_2_ atmosphere at 37°C overnight. The nuclear matrix was extracted from cells according to the method of He et al. [84]. Briefly, cells were incubated with MNNG (50 µM) or ETO (50 µM) for 3 h, washed with PBS, treated with cytoskeleton (CSK) buffer (10 mM PIPES pH6.8, 100 mM NaCl, 300 mM sucrose, 3 mM MgCl_2_, 1 mM EGTA, 0.5% Triton-X-100) for 3 min at 4°C, and subjected to centrifugation at 360×g for 5 min. The pellets were treated with extract buffer (10 mM PIPES pH 6.8, 250 mM ammonium sulfates, 300 mM sucrose, 3 mM MgCl_2_, 1 mM EGTA, 0.5% Triton-X-100) for 5 min at 4°C and centrifuged at 20,000×g for 10 min. They were then treated with CSK buffer B (10 mM PIPES pH6.8, 50 mM NaCl, 300 mM sucrose, 3 mM MgCl_2_, 1 mM EGTA, 0.5% Triton-X-100, 120 KU/ml DNase I) for 60 min at 25°C and centrifuged at 20,000×g for 10 min, then treated with CSK buffer B (10 mM PIPES pH6.8, 100 mM NaCl, 300 mM sucrose, 3 mM MgCl_2_, 1 mM EGTA, 0.5% Triton-X-100, 20 µg/ml RNase A) for 30 min at 25°C and centrifuged at 20,000×g for 10 min. Finally, the pellets were lysed with RIPA buffer. The supernatant was mixed with an equal volume of SDS sample buffer (0.125 M Tris-HCl, 4% SDS, 20% glycerol), subjected to SDS-PAGE, transferred to Immobilon-P membranes (0.45 µm, Merck KGaA), and immunoblotted with anti-A3BGFP antibody (1:2000, ab290, Abcam) and anti-rabbit IgG-HRP (1:10,000, A6154, Sigma-Aldrich). Bands were detected using Immobilon Crescendo HRP Substrate (Merck KGaA) and Fuji medical X-ray film (FUJIFILM Co., Tokyo).

### Immunoprecipitation and DIA analysis

The results of immunoprecipitation and DIA analysis for IFL3, IFL2, XRCC1, XRCC5, XRCC6, and PRKDC are illustrated in Figure 6.

## Supplementary material

online supplementary material 1

## Data Availability

The mass spectrometry data of this study were deposited into the jPOSTrepo (Japan ProteOme STandard Repository) and are available at the following URL: https://repository.jpostdb.org/ under accession number JPST003516.

## Abbreviations

APOBEC3: apolipoprotein B mRNA editing catalytic subunit 3
ARP: aldehyde reactive probe
Act D: actinomycin D
CPT: camptothecin
DDR: DNA damage response
DFC: dense fibrillar component
ETO: etoposide
FC: fibrillarcenter
GC: granular component
IP-MS DIA: immunoprecipitation-mass spectrometry with data-independent acquisition
MNNG: 1-methyl-3-nitro-1-nitrosoguanidine
MPA: mycophenolic acid
PFA: paraformaldehyde
6-TG: 6-thioguanine
ss-DNA: single-stranded DNA
