# Supplementary material for: RNase H-sensitive accumulation of APOBEC3B in a nucleolus after DNA damage
Source: Biosci Rep. 2025 Oct 30;45(10):649–71. doi: 10.1042/BSR20253880 (PMC12784350; doi:10.1042/BSR20253880)
Supplement: online supplementary material 1 [file bsr-45-10-BSR20253880-s001.docx]

**Supplemental figure legends**

**S-Figure 1**

Both hnRNPC1/2 and A3B disappeared from the nucleus after treatment with RNase A.

(A, C) A3B-AcGFP-expressing cells (green) were permeabilized with 0.5% Triton-X-100 and treated with either RNase A or DNase I. The cells were stained with an anti-hnRNP C1/2 antibody (magenta), Nucleolus Bright Red (red), and HOECHST (blue). Merged images are shown as indicated. (B) Quantification of hnRNPC1/2 fluorescence in the nuclei in S-Figure 1A. (D) A3B-AcGFP fluorescence in the nucleus in S-Figure 1C was quantified.

**S-Figure 2**

A3B live-cell imaging and 3D images.

(A) Living cells expressing A3B-AcGFP were imaged for A3B fluorescence 15 min after adding Act D (1 µM), MNNG (50 µM), or CPT (10 µM). Red arrowheads indicate A3B accumulation in nucleolus. (B, C) 3D z-stack confocal images of A3B-AcGFP-expressing cells (green) treated with MNNG (50 µM) for 3 h. The cells were fixed with 4% PFA, permeabilized with 0.5% triton-X 100, and stained with Nucleolus Bright Red (magenta) and HOECHST (blue). Merged images are shown as indicated. Red arrowheads indicate A3B accumulation in nucleolus and white arrowheads indicate nucleolar caps. (C) Fluorescence intensity profiles for A3B (green), Nucleolus Bright Red (magenta), and HOECHST (blue) plotted along the lines indicated in the merged image shown in (B).

**S-Figure 3**

RNase H treatment after methanol fixation did not eliminate A3B accumulation.

(A) A3B-AcGFP-expressing cells (green) were incubated with CPT (1 µM), MNNG (50 µM), or Act D (1 µM) for 3 h and fixed with methanol. Fixed cells were treated with RNase A or RNase H and stained with an anti-DNA/RNA hybrid antibody (S9.6), anti-rabbit IgG conjugated with Ax594, or HOECHST. Arrowheads indicate A3B accumulation. (B) Enlarged image of the dotted-line box in A. Arrowheads indicate A3B-ARP co-accumulation.

**S-Figure 4**

S9.6 signal in nucleolus was attenuated after 5 min of CPT treatment.

A3B-AcGFP-expressing cells (green) were incubated with CPT (1 µM) for the indicated times, permeabilized with 0.1% triton-X 100, and either treated or untreated with RNase A. Cells were fixed with methanol and stained with an anti-DNA/RNA hybrid antibody (S9.6), anti-rabbit IgG conjugated to Ax594, or HOECHST. The S9.6 staining intensity in the nucleolus of each cell without RNase A treatment was quantified. Error bars represent mean ± S. D. (n = 4). Yellow arrowheads indicate S9.6 signal accumulation, and white arrowhead indicates A3B accumulation. ** P < 0.01.

**S-Figure 5**

PFA fixation attenuated S9.6 signaling in the nucleus.

A3B-AcGFP-expressing cells (green) were incubated with CPT (1 µM), MNNG (50 µM), or Act D (1 µM) for 3 h, permeabilized with 0.1% triton-X 100, treated with RNase A or RNase H, fixed with 4% PFA, and stained with anti-DNA/RNA hybrid antibody (S9.6), anti-rabbit IgG conjugated with Ax594, and HOECHST. Yellow and red arrowheads indicate S9.6 accumulation, and white arrowheads indicate A3B accumulation.

**S-Figure 6**

DNA stained with ARP after MNNG treatment.

The cells were incubated with or without MNNG (50 µM) for 3 h. The extracted DNA was air-dried, incubated with ARP for 1 h, blocked with blocking buffer, and stained with SA-Ax647. Fluorescence intensity profiles of ARP (red) and HOECHST (blue) show the distribution of fluorescence across the lines.

**S-Figure 7**

MNNG showed strong accumulation of ARPs in the nucleus.

(A) A3B-AcGFP-expressing cells were incubated with or without MNNG (50 µM) for 3 h. Cells were permeabilized with 0.1% Triton-X-100 for 1 min, treated with or without RNase A, fixed with methanol, blocked with avidin/biotin, incubated with or without ARP for 1 h, blocked with blocking buffer, and stained with streptavidin-Ax647 (red) and HOECHST (blue). (B) Histogram of ARP staining intensity per pixel (35 nm × 35 nm) in the nucleus from the images in A.

**S-Figure 8**

A3B avoided chromosomes.

APOBEC3B-AcGFP-expressing cells (green) were fixed with 4% PFA and stained with anti-hnRNPC1/2 antibody (violet) and HOECHST (blue). The bottom right image shows all merged images.

**S-Figure 9**

MNNG dispersed A3B within the nucleus.

APOBEC3B-AcGFP-expressing HepG2 cells stained with HOECHST for 20 min and then treated with MNNG (50 µM) or ETO (50 µM) for the indicated times on the microscope stage. Nuclear redistribution of A3B was visualized over time.

**S-Figure 10**

After addition of MNNG, A3B entered the nucleolus, but not hnRNPC1/2.

3D z-stack confocal images of A3B-AcGFP-expressing cells (green) treatment with MNNG (50 µM) for 3 h and stained with anti-hnRNPC1/2 antibody (magenta). White arrowheads indicate A3B accumulation in nucleolus.

**S-Figure 11**

MNNG transfers a portion of A3B to the DNA-binding fraction.

APOBEC3B-AcGFP-expressing cells were incubated with MNNG (50 µM) or ETO (50 µM) for 3 h. Cells were sequentially extracted using CSK buffer, extraction buffer, DNase I, and RNase A. Each fraction and the final pellet were solubilized in SDS sample buffer and analyzed by western blot using an anti-GFP antibody.

**S-Figure 12**

RNA helicase DDX17 colocalizes with A3B accumulation following drug addition.

(A) A3B-AcGFP-expressing cells (green) were incubated with Act D (1 µM), CPT (10 µM), or MNNG (50 µM) for 3 h. Fixed and permeabilized cells were incubated with anti-DDX17 antibody, followed by the secondary antibody conjugated with Ax594. The cells were stained with HOECHST for 10 min. The merged images of DDX17 and A3B are shown as indicated. Scale bar = 5 µm. (B) Enlarged image of dotted-line box of A. White arrowheads indicate A3B-DDX17 co-accumulation, and yellow arrowheads indicate Nucleolar caps. The dotted line indicates the boundary of the nucleolus. Scale bar = 2 µm. (C) Fluorescence intensity profiles for DDX17(magenta) and A3B (green) show the distribution of fluorescence along the line indicated in the merged image in (A).

**S-Figure 13**

ILF3 and PRKDC were detected in increased amounts by IP-DIA MS analysis with A3B-AcGFP after DNA damage.

Changes in the quantitative values of ILF3, ILF2, XRCC1, XRCC5, XRCC6, and PRKDC are shown.
